# Molecular Targeted Agent and Immune Checkpoint Inhibitor Co-Loaded Thermosensitive Hydrogel for Synergistic Therapy of Rectal Cancer

**DOI:** 10.3389/fphar.2021.671611

**Published:** 2021-04-16

**Authors:** Huaiyu Zhang, Jiayu Zhang, Yilun Liu, Yang Jiang, Zhongmin Li

**Affiliations:** Department of Gastrointestinal Colorectal and Anal Surgery, China-Japan Union Hospital of Jilin University, Changchun, China

**Keywords:** molecular targeted therapy, immunotherapy, immune checkpoint blockade, thermosensitive hydrogel, cancer treatment, rectal cancer

## Abstract

Molecular targeted therapy has been proved effective in treatment of rectal cancer. Up-regulated expression of programmed death ligand-1 (PD-L1) was observed after the management of molecular targeted therapy, which made the therapeutic effect discounted. Tumors with higher PD-L1 expression were more sensitive and responsive to treatment of PD-L1 inhibitor. Therefore, the combination of molecular targeted therapy and immune checkpoint blockade makes sense. In this study, the copolymers of poly (ethylene glycol)-block-poly (_L_-leucine) (PEG-PLLeu) were synthesized as a thermosensitive hydrogel composite for consecutive release of regorafenib (REG) and BMS202. The mechanical properties of PEG-PLLeu were investigated, confirming that PEG-PLLeu (5 wt.%) was suitable for *in situ* injection as drug-delivery composite at low temperature and stable after sol-gel transition at body temperature. Importantly, the double drug loaded hydrogel showed superior antitumour activity over single drugs in an orthotopic rectal cancer model (CT26-Luc). Further analysis of the tumor tissues suggested that REG upregulated the expression of PD-L1 in tumor tissues. In addition, the immunosuppressive tumor microenvironment of CT26-Luc tumor was distinctly relieved under the effect of BMS202, as characterized by increased infiltration of CD8^+^ T cells in tumors and enhanced secretion of antitumour cytokines (IFN-γ and TNF-α). Moreover, the drug-loaded composite showed no obvious toxicity in histological analysis. Taken together, the administration of REG and BMS202 in the PEG-PLLeu composite could induce a synergistic effect in *in situ* treatment of rectal cancer without obvious toxicity, and thus represented a potential strategy for enhanced *in situ* therapeutic modality.

## Introduction

Globally, rectal cancer is a common malignant tumor with high morbidity and mortality (Bray et al., 2018). So far, surgery remains preferred for therapy in cases of rectal cancer. Besides, chemotherapy and radiotherapy are also the most common methods of cancer treatment including rectal cancer. These adjuvant therapies reduce local recurrence around the tumor bed and suppress distant metastasis after surgical resection. In recent years, preoperative chemoradiotherapy for patients with locally advanced rectal cancer is also an alternative to improve surgical resection rate and sphincter preservation rate (Weiser et al., 2009; Sauer et al., 2012); however, when a rectal tumor deteriorates after conventional treatment, molecular targeted therapy and checkpoint blockade immunotherapy are preeminent candidates for interdicting tumor advancement (Tokumaru et al., 2019; Jung et al., 2020).

Normally, molecular targeted agents mainly take effect in two ways: 1) Monoclonal antibodies competitively bind the ligands involved in tumor angiogenesis or the paired receptors in tumor microenvironment (TME), such as vascular endothelial growth factor (VEGF) and vascular endothelial growth factor receptor (VEGFR), and then interrupt their integration and signal transduction (Hurwitz et al., 2004); 2) Kinase inhibitors interdict the phosphorylation and signal transduction downwards (Wilhelm et al., 2011; Jang et al., 2019). Regorafenib (REG) is a type of multi-kinase inhibitor and has been approved in treatment of advanced colorectal cancer (Skarderud et al., 2018). A primary function of REG is that it blocks the phosphorylation of VEGFR-2 after binding with VEGF in TME, and then influences tumour-induced pathologic angiogenesis (Wilhelm et al., 2011). Once uncontrolled angiogenesis is ceased, the abnormal blood vessels in TME tend to be normalized to cope with sufficient oxygen supply, increased migration of antitumour immune cells, and enhanced penetration of antitumour agents into the tumor which contributed to the suppression thereof; however, it was found that molecular targeted therapy boosted upregulation of programmed death ligand-1 (PD-L1) in tumor tissues (Liu et al., 2015). This immune accommodation of a tumor may hamper the molecular targeted agent-based antitumour efficiency. Therefore, how to counteract immunosuppression involved with PD-L1 up-regulation is key to improving therapeutic responses of molecular targeted therapy. Those types of tumor with higher PD-L1 expression were more sensitive and responsive to treatment of PD-L1 inhibitor (Ribas and Tumeh, 2014; Patel and Kurzrock, 2015). Therefore, the combination of molecular targeted therapy and checkpoint blockade immunotherapy makes sense.

Checkpoint blockade immunotherapy is a burgeoning strategy for cancer solution which targets the T-cell co-inhibitory signaling pathways (Song et al., 2018). Nowadays, several monoclonal antibodies have been approved for immune checkpoint blockade, such as nivolumab (Halmos et al., 2020), pembrolizumab (Halmos et al., 2020), avelumab (Verschraegen et al., 2020), etc. The monoclonal antibodies competitively bind and block PD-1 on T-cells or PD-L1 on tumor cells. This checkpoint blockade immunotherapy neutralizes incapacitation of T cells triggered by pairing of PD-1/PD-L1 and promotes antitumour immunity of tumor infiltrating lymphocytes (TILs). Apart from monoclonal antibodies, small-molecule compounds have also been investigated for use in checkpoint blockade immunotherapy (Bailly and Vergoten, 2020). Compared with anti-PD-1/PD-L1 monoclonal antibodies, the chemical constructions of small-molecule drugs are more stable, which guarantees that they are easy to store. In addition, the cost and selling price of small-molecule drugs is normally low. As a result of that, more patients can be treated with small-molecule anticancer drugs. BMS202 is a type of PD-L1 inhibitor which propels aggregation of PD-L1 on tumor cells into the dimer structure (Zak et al., 2017; Bailly and Vergoten, 2020). This hinders the integration between PD-1 and PD-L1 which shields immunosuppressive signals from tumor cells and enhances recognition and killing from TILs. In this way, BMS202 counteracts immunosuppression triggered by PD-L1 upregulation and promotes antitumour immune responses, thus may boost therapeutic responses of molecular targeted agents.

To address this, we constructed an orthotopic rectal cancer model, and proved that BMS202 would boost tumor responses to treatment of REG. To reduce anticancer drug-associated side-effects and improve treatment efficacy, various polymer-based carriers have been designed by researchers and employed in drug delivery (Ding et al., 2019a; Ding et al., 2019b; Sun et al., 2019; Zheng et al., 2020; Ma et al., 2021). In addition to the popular nanoparticle-based drug carriers (Wang et al., 2018; Chen et al., 2020; Feng et al., 2020; Jiang et al., 2020; Zhang et al., 2020), hydrogels were also designed and optimized in drug delivery nowadays (Guo et al., 2020). In this study, we designed an injectable thermosensitive hydrogel and performed peritumoral injection of the antitumour-agent-loaded hydrogel. Various amino acids are essential nutrients for the body’s metabolism. Therefore, the hydrogel prepared from amino acids as raw materials not only has obvious advantages in biocompatibility, but also can be degraded into neutral products under the action of various proteases in the body. Besides, this process does not produce acidic metabolism to change the pH value of microenvironment at tumor site, which is very important for the treatment of tumors. The _l_-leucine based thermosensitive hydrogel in this study exhibited superior biocompatibility and step-by-step degradation which avoided repeated administration of REG and BMS202. Moreover, the combination of REG and BMS202 resulted in coadjutant antitumour activities with no obvious side-effects. We propose that the combination of locally applied molecular targeted therapy and checkpoint blockade immunotherapy may be meaningful as a therapy for rectal cancer.

## Materials and Methods

### Materials

The amino-terminated poly (ethylene glycol) (mPEG_45_-NH_2_) was synthesized through the protocol described in our previous work (Chen et al., 2015). _L_-Leucine *N*-carboxyanhydride (_L_-Leu NCA) was obtained from Chengdu Enlai Biological Technology Co., Ltd. (Chengdu, China). Penicillin, streptomycin, trypsin-EDTA (0.05% trypsin and 0.02% EDTA) solution, RPMI 1640 medium, and new-born calf serum (NBCS) were bought from Gibco (Grand Island, NY, United States). Toluene, N,N-dimethylformamide (DMF; anhydrous), diethyl ether, elastase, and chymotrypsin were obtained from Aladdin Reagent Co., Ltd. (Shanghai, China). Hematoxylin and eosin (H&E) staining solution was purchased from Sigma-Aldrich (Shanghai, China). BMS202 was purchased from Selleck Chemicals (United States). PD-L1 antibody and Phospho-VEGF Receptor 2 antibody used for western blot (WB) were purchased from eBioscience (San Diego, CA, United States). PE-cy7-anti-CD45, FITC-anti-CD3, PerCP-cy5.5-anti-CD4, and Pacific Blue-anti-CD8 used for flow cytometry were purchased from eBioscience (San Diego, CA, United States). Enzyme-linked immunosorbent assay (ELISA) kits were purchased from Shanghai Lengton Bioscience Co., Ltd. (Shanghai, China). All the other chemicals were purchased from Beijing Chemical Industry Group Co., Ltd. (China).

### Preparation of Poly (Ethylene Glycol)-Block-Poly (_L_-Leucine) Copolymers

The PEG-PLLeu copolymers were prepared by the ring-opening polymerization (ROP) of _L_-Leu NCA initiated by mPEG_45_-NH_2_ (Ding et al., 2015; Liu et al., 2020). In brief, 2.0 g of mPEG_45_-NH_2_ was added into toluene and dewatered by azeotropic distillation. The anhydrous mPEG_45_-NH_2_ was dissolved in 45.0 ml of anhydrous DMF. After that, 2.78 g of _L_-Leu NCA was added and stirred with the solution. After reaction for 3 days at room temperature and under an N_2_ atmosphere, the resulting solution was placed into 500 ml of diethyl ether by pre-freezing, and the precipitate was then collected. The collected precipitate was then dissolved in anhydrous DMF and dialyzed against water. The final products were obtained by lyophilisation.

### Characterization of Poly (Ethylene Glycol)-Block-Poly (_L_-Leucine) Copolymers

The chemical structure of PEG-PLLeu copolymers was investigated by using proton nuclear magnetic resonance (^1^H NMR) and Fourier-transform infrared (FT-IR) spectra as in our previous study (Ding et al., 2015). Typical ^1^H NMR spectra of PEG-PLLeu copolymers dissolved in deuterated trifluoroacetic acid (TFA-*d*) were determined on a Bruker AV 400 NMR spectrometer (Ettlingen, Germany). According to the potassium bromide (KBr) method, FT-IR spectra of the PEG-PLLeu copolymers were detected on a Bio-Rad Win-IR instrument (Cambridge, MA, United States).

### Phase Diagrams

The phase diagrams of the polymers were determined by a test tube inverting method. In brief, these polymers were dissolved in PBS with a concentration of 3–7 wt.% and stirred for 24 h on an ice bath. Next, 200.0 μL of each sample with the given concentration was added to a 2-ml cylindrical vial and transferred into a thermostat at the pre-set temperature. The temperature rise was programmed to be 2°C per 10 min. The temperature corresponding to the sol–gel transition when that at which no liquid flow was noticed within 30 s after inverting the cylindrical vial.

### Rheological Analyses

Dynamic mechanical characteristics of the PEG-PLLeu copolymers were investigated by rheological analyses on an MCR 301 Rheometer (Anton Paar, GmbH, Germany). The pre-set temperature interval was between 5 and 60°C, and the temperature was increased at 0.5°C/min. Samples dissolved in PBS (350.0 μL) were titrated onto a 25.0 mm sample-maintained plate with a gap of 0.5 mm between the plates, and maintained for 5 min before structural recovery. Meanwhile, a thin layer of silicone oil was dropwise dripped onto the edge of the samples for evaporation limitation. The final data pertaining to storage modulus (*G*′) and loss modulus (*G*″) were collected under a strain and frequency of 1% and 1 rad s^−1^.

### Scanning Electron Microscopy

The morphology of PEG-PLLeu copolymers was identified by SEM examination which was performed on a Philips XL30 instrument (Eindhoven, Netherlands). In brief, the PEG-PLLeu copolymers were dissolved in phosphate-buffered saline (PBS) and stirred for 24 h on an ice bath. Thereafter, the samples were placed in a thermostatically controlled water bath for 10 min at the pre-set temperature of 37°C to allow gel formation. Then, the samples were frozen in liquid nitrogen for 30 s before lyophilization under vacuum. Finally, the cross sections of lyophilized samples were investigated by SEM under an acceleration voltage of 10 kV.

### *In Vitro* Degradation

Briefly, the PEG-PLLeu polymers were premixed in PBS (5 wt.%) and stirred for 24 h on an ice bath. After that, 0.5 ml of the copolymer solution was added to each 3-ml tube whose masses were recorded beforehand. The solution was then located into a thermostat for 12 min with the pre-set temperature of 37°C for gel formation. The mass of the gel loaded tube was recorded again. Then, 2.0 ml of PBS with or without elastase or chymotrypsin was dripped to the surface of the gel, and the glass tube was transferred into a shaking incubator at 37°C and 70 rpm. The supernatant was removed every day before accurately weighing the gel-loaded tube. After that, an equal dosage of fresh solution was supplemented for the next measurement. The mass loss of the PEG-PLLeu gel was monitored for 30 days, and the data were used to analyze the degradation.

### Cell Line and Animal Model Establishment

CT26-Luc cell line stably expressing luciferase was purchased from Golden Tran Co., Ltd. (Changchun, China). Four-week-old female BALB/c mice (18–20 g) were obtained from Vital River Laboratory Animal Center (Beijing, China). Animals used in this study were fed and handled following the guidelines of the Institutional Animal Care and Use Committee of Jilin University. For establishment of the orthotopic rectal tumor model, mice were subjected to fasting for 12 h and anesthetized using 2% pentobarbital sodium. The mice were bodily fixed in a supine position. The anal tube was exposed by hook tweezers. CT26-Luc cells (1 × 10^5^ cells in 50 µL of PBS) were submucosally injected into the posterior wall of the rectum with an insulin-gauge syringe. The puncture site was approximately 2 mm proximal to the anal edge. Light pressure was exerted at the puncture site to avoid leakage of the cell suspensions. Tumor formation and burden were monitored through bioluminescent detection using BRUKER Xtreme II. Under anesthesia, the bioluminescent detection was conducted 10 min after intraperitoneal (i.p*.*) injection of 100 µL solution of D-luciferin (10 mg ml^−1^) (Song et al., 2018).

### *In Vivo* Antitumour Assay

Two weeks after inoculation of CT26-Luc cells, the tumour-bearing mice were subjected to the first detection of bioluminescence, and randomly allocated into different treatment groups (*n* = 5). The PEG-PLLeu copolymers were then prepared into injectable gel with or without antitumour agents and maintained in an ice bath. After that, CT26-Luc tumour-bearing mice were given an *in situ* pericarcinomatous injection of 100 μL PBS, Gel, Gel/REG, Gel/BMS, and Gel/(BMS + REG) c. CT26-Luc tumor burdens were monitored through bioluminescent detection using BRUKER Xtreme II every 5 days. The enlargement of tumor burdens was calculated as bioluminescent intensities over the initial values thereof. Tumor suppression rate (TSR %) = (*I*_c_ − *I*_x_)/*I*_c_ × 100%, where *I*_c_ represents the bioluminescent intensity of the PBS group, and *I*_x_ denotes the bioluminescent intensity of other treatment groups. The body mass of all groups was recorded every other day. Mice in different groups were sacrificed at the given time points. Tumors were obtained for immunohistochemistry staining, flow cytometry analysis, ELISA, and WB assay. Spleens and sentinel lymph nodes were obtained for flow cytometry analysis. Major organs (heart, liver, spleen, lung, and kidney) were obtained for H&E staining.

### Histopathological and Immunohistochemistry Analyses

The mice were sacrificed the next day after the last bioluminescent detection. After collection of the tumor tissues or organs, the samples were immersed into 4% (W/V) PBS-buffered paraformaldehyde for 12 h and embedded in paraffin. Then the paraffin-embedded samples were sliced for further H&E staining and immunohistochemistry analyses (Ki-67 and caspase-3). The H&E staining and immunohistochemistry results were investigated using a microscope (Nikon Eclipse Ti, Optical Apparatus Co., Ardmore, PA, United States).

### Flow Cytometry Assay

Briefly, tissues of tumor, sentinel lymph nodes, and spleen were harvested and digested with lysate buffer (collagenase A and dnaase) at 37°C for 40 min. After the digestion of ACK buffer, lysis of red blood cells was completed, then, the remaining cells were obtained and fabricated into single-cell suspensions. After staining with fluorescence conjugated antibodies, disparate cells in single-cell suspensions were fixed with 4% PFA and quantitatively analyzed via fluorescent-activated cell sorting (FACS).

### Western Blot Analysis

Tumor tissues of different groups were collected after sacrificing the mice. The samples were then prepared and lyzed to obtain the whole protein. After centrifugation, quantification, and boiling for 10 min, samples were loaded onto 12% SDS-PAGE gel and electrophoretically transferred to PVDF membranes. The membranes were then blocked with 5% (W/V) bovine serum albumin in Tris-buffered saline plus 0.1% Tween 20. After incubating with primary antibody on a shaker at 4°C overnight, the membranes were washed, incubated for 1 h with a secondary antibody. Finally, after washing to remove superfluous antibodies, the proteins then detected were visualized (GAPDH was utilized as an internal reference).

### Enzyme-Linked Immunosorbent Assay Detection

Cytokine analysis including IFN-γ and TNF-α was performed using the corresponding ELISA kits (Shanghai Lengton Bioscience Co., Ltd., Shanghai, China) according to the standard protocols provided by the manufacturer.

### Statistical Analyses

Data analyzed in this manuscript are presented as means ± standard deviations. Differences between each group were determined by Student’s t-test with SPSS 17.0 (SPSS Inc., Chicago, IL, United States). Statistical significance was defined as *p < 0.05, and **p < 0.01 and ***p < 0.001 were regarded as highly significant.

## Results and Discussion

### Preparation and Characterization of Poly (Ethylene Glycol)-Block-Poly (_L_-Leucine) Copolymers

**FIGURE 1:**
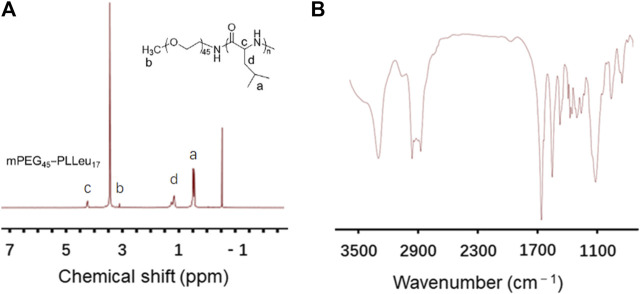
Characterization of PEG-PLLeu copolymers. **(A)** ^1^H NMR spectra of PEG-PLLeu copolymers. **(B)** FTIR detection of PEG-PLLeu copolymers.

The PEG-PLLeu copolymers in this study were prepared by ROP of _L_-Leu NCA initiated by mPEG_45_-NH_2_ as mentioned. ^1^H NMR and FTIR detections were employed for analyzing the chemical structure of PEG-PLLeu. As shown in Figure 1A, all the peak signals of protons in PEG and polymerized _L_-Leu block were assigned in the ^1^H NMR spectra, which illustrated the successful manufacture of PEG-PLLeu copolymers. The degrees of polymerization (DPs) of _L_-Leu units in PEG-PLLeu copolymer block were analyzed based on the integrated area assigned to the side methyl protons (-CH_2_CH(CH_3_)_2_) and that assigned to the methylene proton in PEG: the calculated DPs value of _L_-Leu units in PEG-PLLeu copolymers was 17. As displayed in Figure 1B, the outcome of FTIR spectra also proved the successful generation of _L_-Leu blocks based on the appearance of the typical amide bands at 1,548 cm^−1^ (υ_C(O)-NH_) and 1,653 cm^−1^ (υ_C=O_).

**FIGURE 2:**
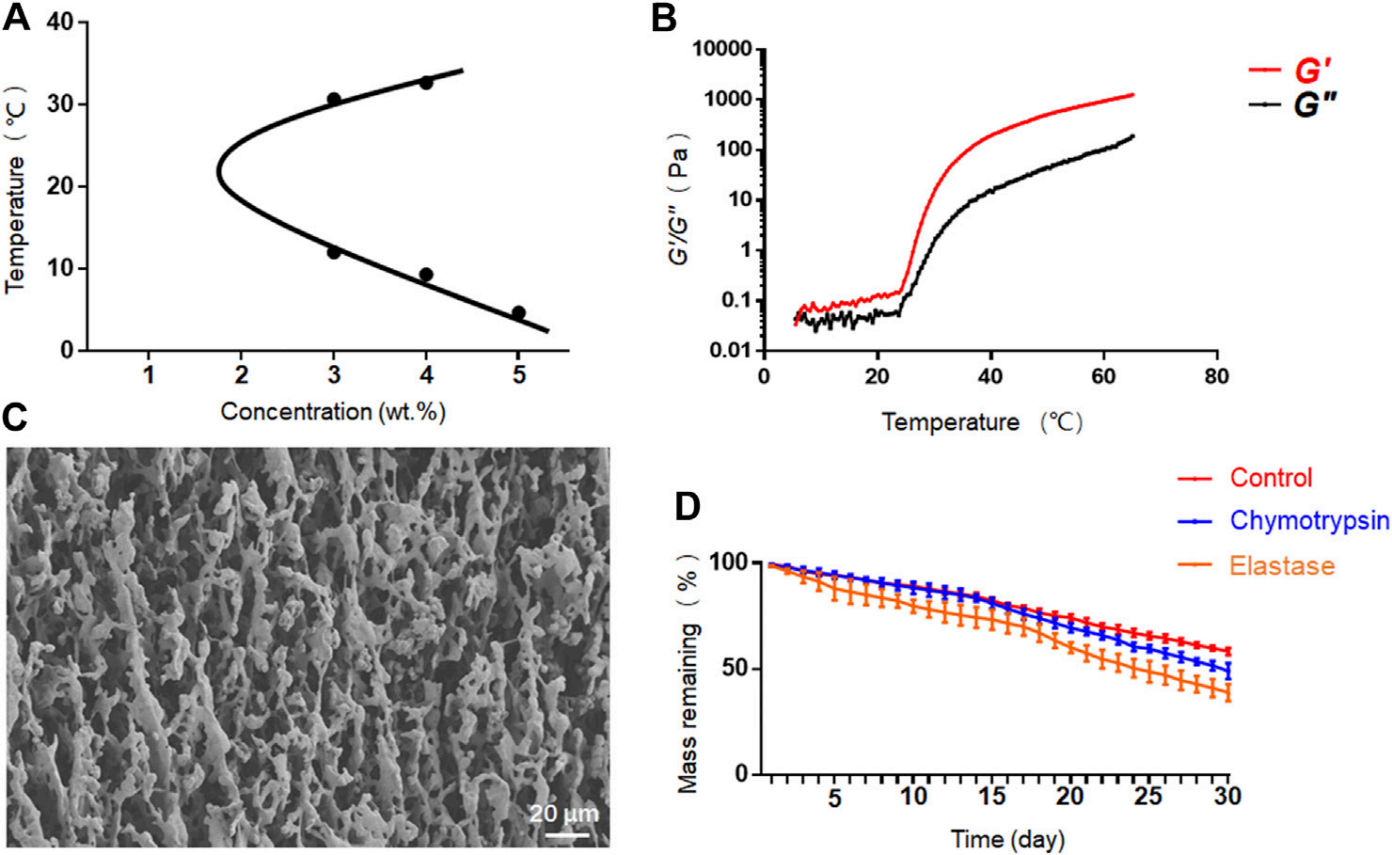
Characterization of PEG-PLLeu thermogels. **(A)** Phase diagram of PEG-PLLeu in PBS solution at concentrations of 3.0–7.0 wt.%. **(B)** Rheological analysis of PEG-PLLeu thermogels (5.0 wt.%). **(C)** SEM images of PEG-PLLeu thermogels (5.0 wt.%). **(D)** *In vitro* mass-remaining profiles of PEG-PLLeu thermogels incubated in PBS with or without elastase or chymotrypsin.

We further investigated the phase diagram of PEG-PLLeu in PBS solution at concentrations of 3.0–7.0 wt.%. As shown in Figure 2A, phase transition appeared only at concentrations of 3.0–5.0 wt.%. The average sol-gel transition temperatures were 12.00/30.67°C at 3.0 wt.% and 9.33/32.67°C at 4.0 wt.%, suggesting that PEG-PLLeu at these concentrations would not gelatinize at body temperature. At the concentration of 5.0 wt.%, PEG-PLLeu copolymer gelatinized at 4.67°C and was stable as the temperature increased. Herein, 5.0 wt.% PEG-PLLeu was suitable for *in situ* injection as drug container when manipulation at low temperature was guaranteed. This screened concentration (5.0 wt.%) was chosen for all further characterisations.

Rheological analysis of PEG-PLLeu was then conducted in which *G*′ referred to the stiffness of PEG-PLLeu and *G*″ represented its viscosity. As shown in Figure 2B, the value of *G*″ exceeded *G*′ below 6°C, and then *G*′ exceeded *G*″ as the temperature increased, implying the gelation of PEG-PLLeu. This outcome was consistent with that inference drawn from the phase diagram. Besides, the maximum *G*′ value of PEG-PLLeu hydrogel exceeded 1,200 Pa, indicating the proper mechanical intensity of PEG-PLLeu hydrogel as an *in situ* drug release composite (Zhang et al., 2018). The microscopic morphology of PEG-PLLeu cross section was further investigated by SEM detection: the outcome revealed an interconnected porous microstructure of the PEG-PLLeu hydrogel (Figure 2C).

The favourable biodegradability is a vital factor for biomedical application of various thermogels. The *in vitro* degradation behavior of PEG-PLLeu thermogel was investigated in PBS with or without elastase or chymotrypsin over a 30-day period. As displayed in Figure 2D, degradation of PEG-PLLeu thermogels was faster in PBS with elastase or chymotrypsin than that in pure PBS due to the enzymolysis of polypeptides by elastase and chymotrypsin. This degradation property of PEG-PLLeu thermogel exhibited suitable biodegradability kinetics and could afford the long-term effect of the loading agents.

### Antitumour Performance *In Vivo*

**FIGURE 3:**
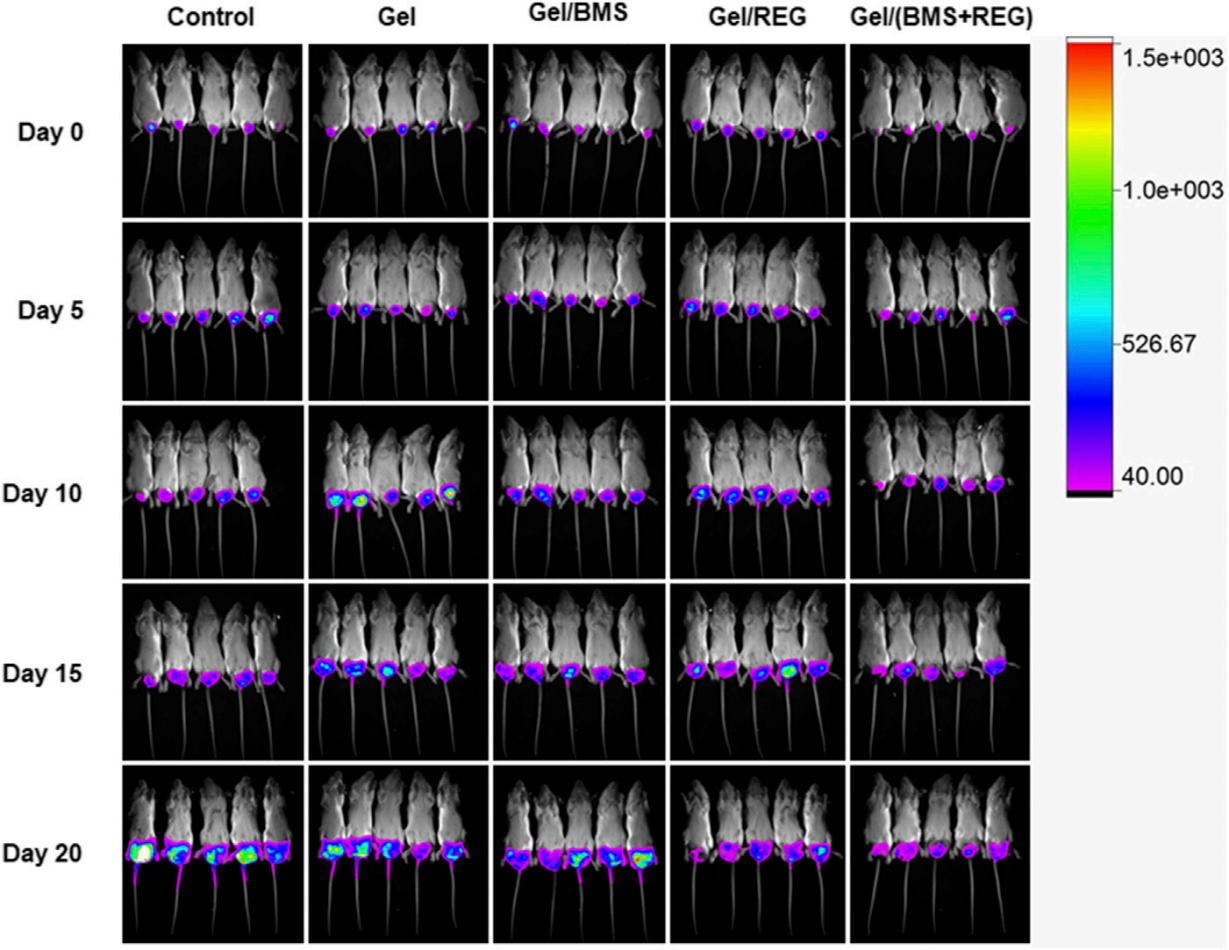
Bioluminescent detection of the PBS, Gel, Gel/REG, Gel/BMS, and Gel/(BMS + REG) groups, from the day before treatment to 20 days after *in situ* treatment.

**FIGURE 4:**
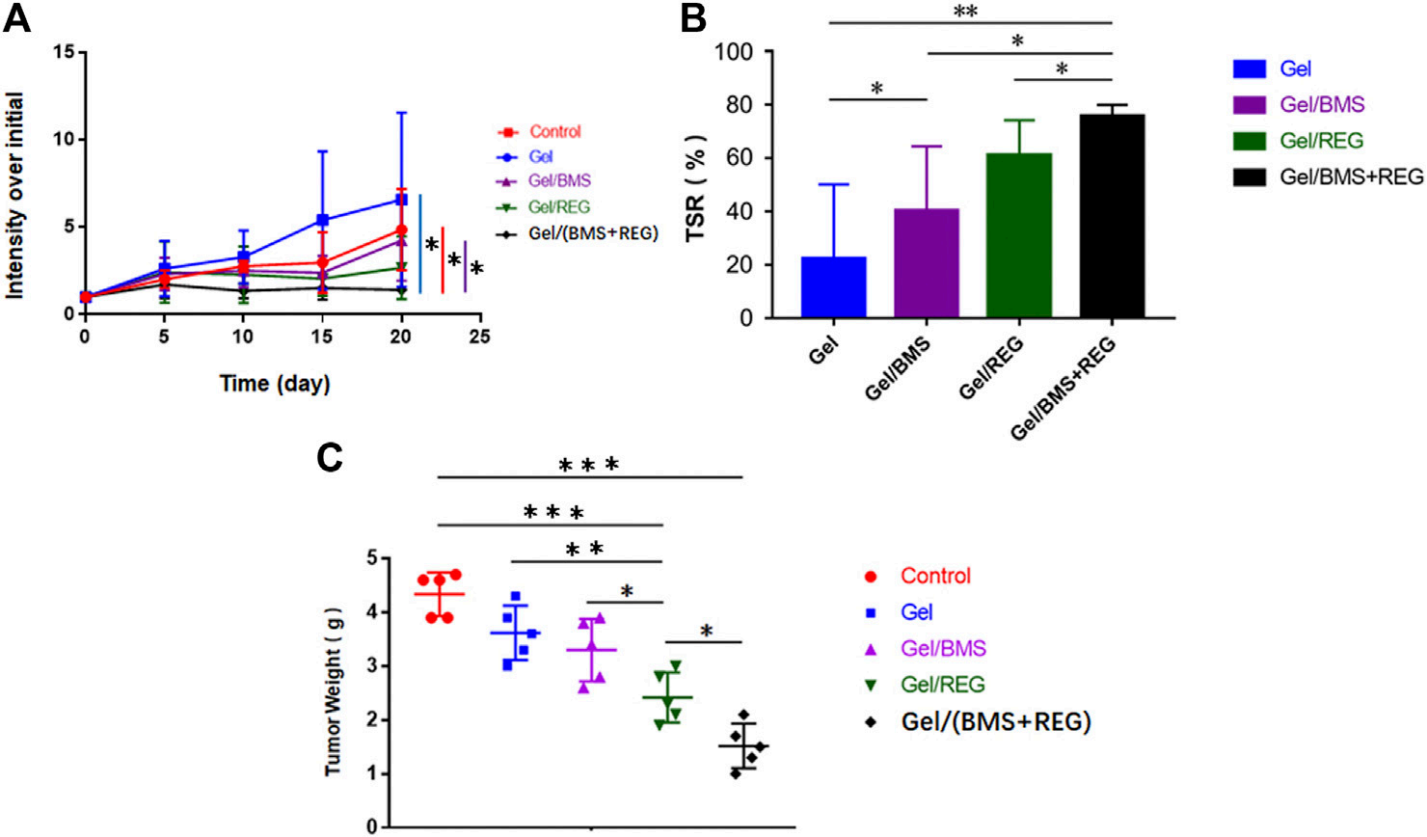
*In vivo* anticancer efficacy of different groups (REG: 45 mg/kg, BMS202: 50 mg/kg). **(A)** Tumor growth curves of orthotopic CT26-Luc tumors in PBS, Gel, Gel/REG, Gel/BMS, and Gel/(BMS + REG) groups (*n* = 5). **(B)** TSR% results on day 20. **(C)** Tumor masses of different groups. Data are represented as the mean ± SD (*p < 0.05, **p < 0.01, and ***p < 0.001).

Regarding the unique anti-angiogenic mechanism of REG and immune regulation property of BMS202, we directly conducted the antitumour assay *in vivo*. Besides, in the light of the various properties of PEG-PLLeu thermogel, 5.0 wt.% PEG-PLLeu was programmed to be carrier of REG and/or BMS202 for the *in vivo* inhibition against CT26-Luc tumor. BALB/c mice first received inoculation of CT26-Luc cells. Two weeks after the inoculation, the inoculation-accepted mice were subjected to bioluminescence, and randomly allocated into different treatment groups. Mice of different groups were then subjected to pericarcinomatous injection with PBS, Gel, Gel/REG, Gel/BMS, and Gel/(BMS + REG), and bioluminescent detection was conducted every 5 days until 20 days after the treatment (Figure 3). In order to investigate the anticancer efficacy of each group, we draw a curve of tumor inhibition on the basis of bioluminescent detection. As shown in Figure 4A, Gel/REG showed potent tumor inhibition. On the contrary, the treatment efficacy of Gel/BMS was not obvious. However, there was the most significant tumor inhibition observed in Gel/(BMS + REG) treatment group. This indicated that BMS202, as a PD-L1 inhibitor, was synergetic with molecular targeted agent such as REG. But the treatment efficacy of BMS202 alone was quite limited. Apart of that, TSR% of Gel, Gel/REG, Gel/BMS, and Gel/(BMS + REG) on day 20 were 22.33 ± 27.79, 61.09 ± 13.07, 40.38 ± 23.97, and 75.76 ± 4.21%, respectively, implying the synergistic suppressive efficiency of combining molecular targeted agent and checkpoint inhibitor in CT26-Luc rectal cancer therapy (Figure 4B). Moreover, with the consideration of deviation relevant to bioluminescent detection, the whole tumors of each mouse were obtained and compared on day 21 after sacrificing the mice. Tumor masses of PBS, Gel, Gel/REG, Gel/BMS, and Gel/(BMS + REG) groups were 4.34 ± 0.40, 3.62 ± 0.51, 2.42 ± 0.47, 3.30 ± 0.58, and 1.52 ± 0.41 g, respectively (Figure 4C). Obviously, tumor masses in the Gel/(BMS + REG) group were significantly lower than those in the other treatment groups, consistent with the bioluminescent analysis.

**FIGURE 5:**
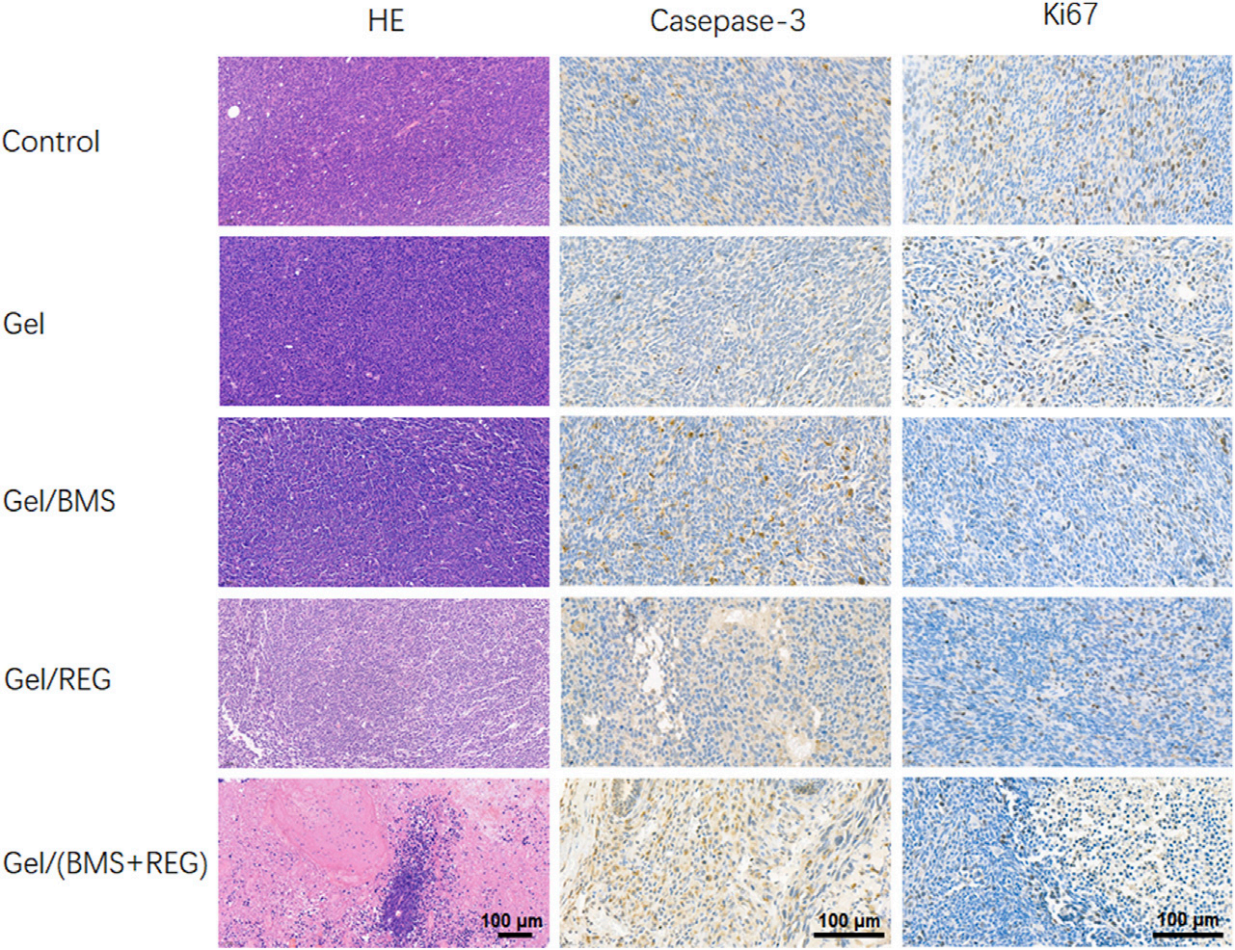
H&E staining and immunohistochemistry analysis of tumor tissues.

H&E staining of tumor tissues and immunohistochemistry staining of caspase-3 and ki67 were then performed to demonstrate the histological variation, apoptosis, and proliferation of CT26-Luc tumors in various groups. As shown in Figure 5, single application of REG could achieve considerable therapeutic outcomes and was more efficient in terms of tumor inhibition compared with BMS202. On the contrary, single utilization of BMS202 was slightly influential in apoptosis and proliferation of CT26-Luc cells, with minor morphological changes to CT26-Luc cells via H&E staining; however, there were the significant cytological variation of H&E slides, the highest caspase-3 expression, and the lowest ki67 expression in Gel/(BMS + REG) group, matching the therapeutic efficiency of this synergistic style of treatment.

**FIGURE 6:**
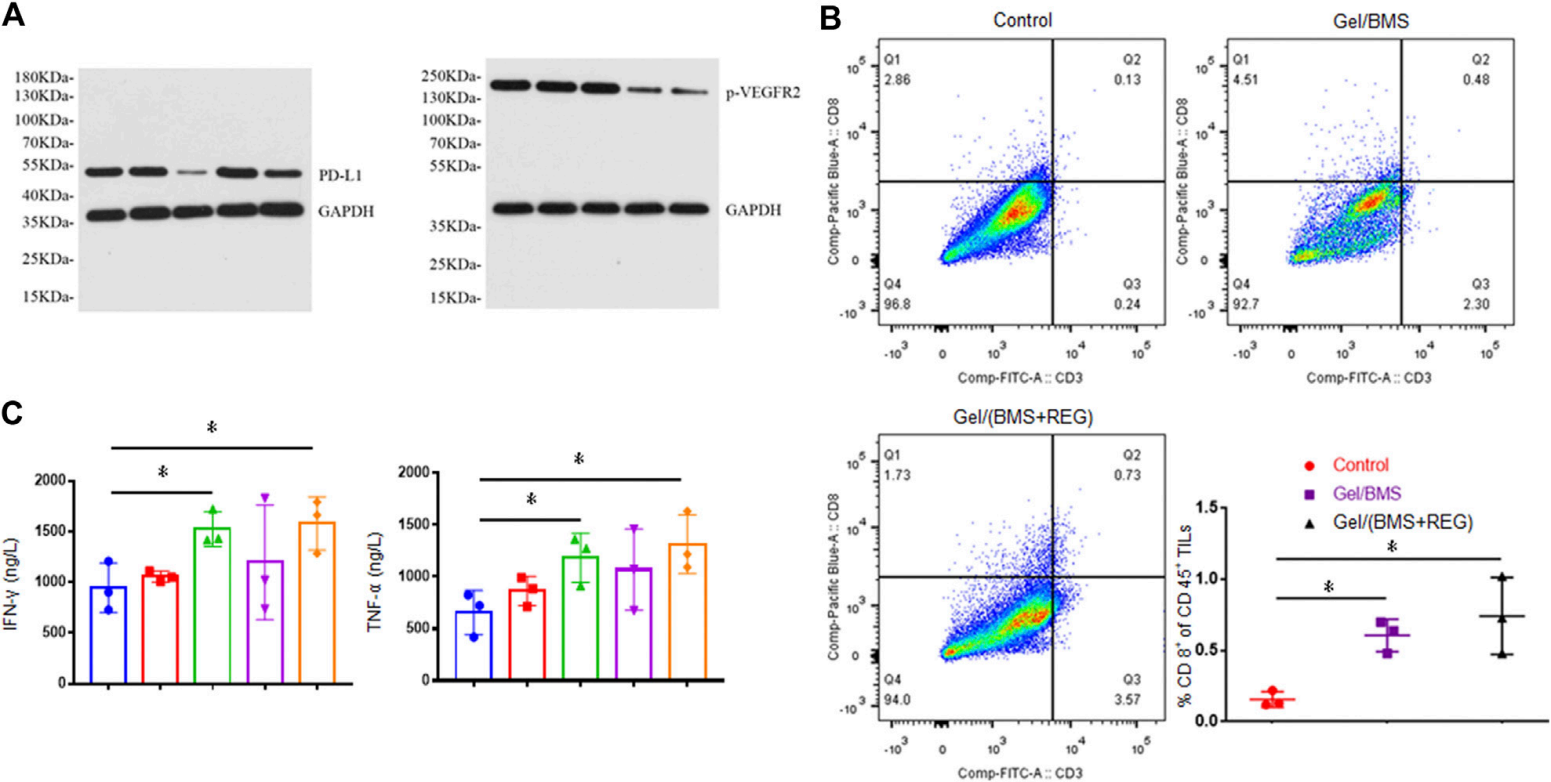
WB assay, flow cytometry, and ELISA detection of tumor tissues. **(A)** Expression of PD-L1 and p-VEGFR2 in CT26-Luc tumor tissues in all groups. From left to right: PBS, Gel, Gel/BMS, Gel/REG, and Gel/(BMS + REG) groups. **(B)** Flow cytometry analysis of CD8^+^ T cell ratio in TILs of PBS, Gel/BMS, and Gel/(BMS + REG) groups (*n* = 3). **(C)** The excretive levels of Th1-type cytokines IFN-γ and TNF-α in PBS, Gel, Gel/BMS, Gel/REG, and Gel/(BMS + REG) groups (*n* = 3). From left to right: PBS, Gel, Gel/BMS, Gel/REG, and Gel/(BMS + REG) groups. Data are represented as the mean ± SD (*p < 0.05).

Researchers found that the types of tumors with higher PD-L1 expression were more sensitive and responsive to treatment of PD-L1 inhibitor (Ribas and Tumeh, 2014; Patel and Kurzrock, 2015). Besides, tumor tissue of renal cell carcinoma patients treated with molecular targeted therapy was found to display increased PD-L1 expression (Liu et al., 2015). Herein, WB assay was performed in this study to verify the expression of PD-L1 in CT26-Luc tumor influenced by treatment of REG. As shown in Figure 6A, tumor samples in the Gel/BMS group displayed the lowest expression of PD-L1. By contrast, the samples in the Gel/REG group displayed the highest expression of PD-L1. Moreover, PD-L1 expression of Gel/(BMS + REG) group was notably up-regulated compared with that of Gel/BMS group, which reflected the effect of REG treatment on PD-L1 expression of CT26-Luc tumors. The expression of phosphorylated VEGFR-2 (p-VEGFR2) was also examined via WB, which demonstrated the prime anti-angiogenic mechanism of REG.

To further explore whether BMS202 relieved the deteriorative TME involved with PD-L1 upregulation and its potential mechanism, we conducted flow cytometry assay and examined the CD8^+^ T cell in TILs. As shown in Figure 6B, treatment including BMS202 significantly improved recruitment of CD8^+^ T cell into tumors, fulfilling their mission of tumor cell elimination. The ratio of CD8^+^ T cell in spleen and tumour-draining lymph nodes was also examined, and no significant difference was discovered between Gel/BMS, Gel/(BMS + REG), and the control group (Supplementary Figures S1, S2). This indicated that *in situ* application of BMS202 only relieved the immunological condition of local TME. The variational secretion of IFN-γ and TNF-α in TME is always relevant to the recruitment of CD8^+^ T cell. IFN-γ and TNF-α not only inhibit the growth of tumor cells directly, they also promote recognizing and killing of tumor cells from tumour-associated antigen-specific CD8^+^ T cells. As a result, excretive levels of Th1-type cytokines IFN-γ and TNF-α were further detected via ELISA assay. As shown in Figure 6C, the utilization of BMS202 specifically boosted secretion of IFN-γ and TNF-α and revoked the immunosuppressive TME of CT26-Luc tumor, thus resulting in the activation of CD8^+^ T cells and consequently enhanced efficacy of this synergistic therapy.

### Safety Assessment of Poly (Ethylene Glycol)-Block-Poly (_L_-Leucine) Copolymers *In Vivo*

**FIGURE 7:**
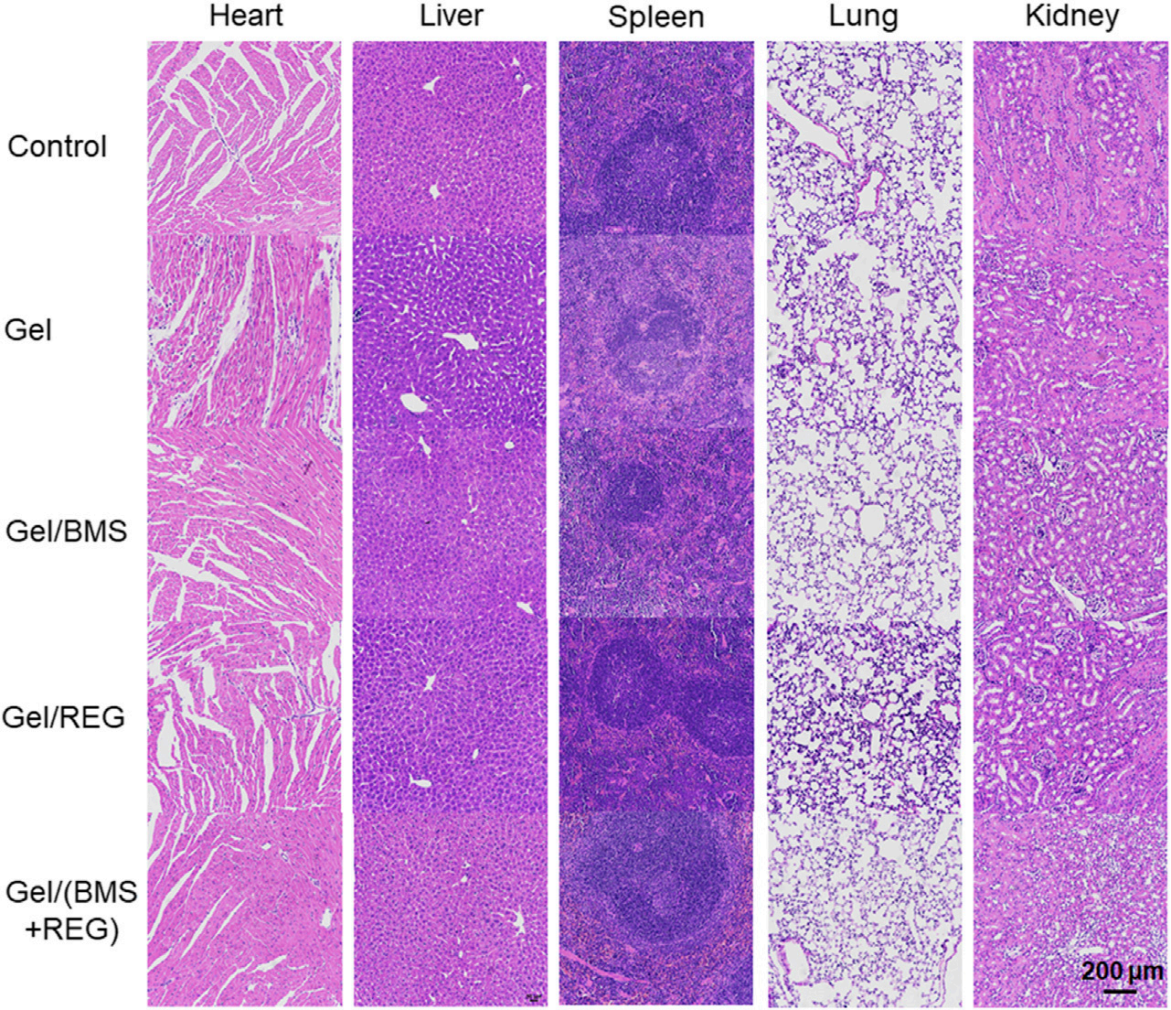
Representative images of H&E staining for major organs obtained from mice in various treatment groups.

As a promising drug delivery system for *in situ* therapy of rectal cancer, the systematic toxicity and safety of the PEG-PLLeu thermogel is a primary concern. Besides, toxicity of REG and BMS202 should also to be monitored. We supervised specific parameters in mice as safety indexes. As shown in Supplementary Figure S3, the body mass of mice in all groups was monitored, and no obvious loss of mass was observed between each group, suggesting that implantation of the PEG-PLLeu thermogel with or without REG/BMS202 did not influence the overall health of the mice. H&E staining and analysis was further introduced to investigate the potential histopathological microlesions. As shown in Figure 7, no prominent histopathological abnormalities or microlesions were noticed in the main organs. Overall, results in this study demonstrated the potential use of the REG/BMS202-loaded PEG-PLLeu thermogel as an efficient drug delivery system for synergistic treatment of rectal malignancies while alleviating the adverse effects thereof.

## Conclusion

In summary, the PEG-PLLeu thermogels were successfully prepared by ROP. The thermogels displayed favourable mechanical properties and biodegradability because of the unique properties of the polypeptide copolymers. In addition, the local delivery system for *in situ* release of REG and BMS202 facilitated synergetic suppression on orthotopic CT26-Luc rectal tumors. Regardless of the upregulation of PD-L1 attributed to REG, the immunosuppressive TME of CT26-Luc tumor was distinctly relieved of the effects of BMS202, characterized by the increased infiltration of CD8^+^ T cell in CT26-Luc tumor and enhanced secretion of antitumour cytokines (IFN-γ and TNF-α). Moreover, the drug delivery composite exhibited no obvious toxicity in histopathological analysis. Taken together, the allied administration of molecular targeted therapy and checkpoint blockade immunotherapy by virtue of PEG-PLLeu hydrogel represented a potential strategy for enhanced *in situ* therapeutic modality for rectal cancer.

## Data Availability

The original contributions presented in the study are included in the article/Supplementary Material, further inquiries can be directed to the corresponding authors.

## References

[B1] Bailly C. Vergoten G. (2020). Protein homodimer sequestration with small molecules: focus on PD-L1. Biochem. Pharmacol. 174, 113821. 10.1016/j.bcp.2020.113821 31972166

[B2] Bray F. Ferlay J. Soerjomataram I. Siegel R. L. Torre L. A. Jemal A. (2018). Global cancer statistics 2018: GLOBOCAN estimates of incidence and mortality worldwide for 36 cancers in 185 countries. CA: A Cancer J. Clinicians 68 (6), 394–424. 10.3322/caac.21492 30207593

[B3] Chen J. Ding J. Zhang Y. Xiao C. Zhuang X. Chen X. (2015). Polyion complex micelles with gradient pH-sensitivity for adjustable intracellular drug delivery. Polym. Chem. 6 (3), 397–405. 10.1039/c4py01149j

[B4] Chen J. Jiang Z. Xu W. Sun T. Zhuang X. Ding J. (2020). Spatiotemporally targeted nanomedicine overcomes hypoxia-induced drug resistance of tumor cells after disrupting neovasculature. Nano Lett. 20 (8), 6191–6198. 10.1021/acs.nanolett.0c02515 32697585

[B5] Ding J. Chen J. Gao L. Jiang Z. Zhang Y. Li M. (2019a). Engineered nanomedicines with enhanced tumor penetration. Nano Today 29, 100800. 10.1016/j.nantod.2019.100800

[B6] Ding J. Feng X. Jiang Z. Xu W. Guo H. Zhuang X. (2019b). Polymer-mediated penetration-independent cancer therapy. Biomacromolecules 20 (12), 4258–4271. 10.1021/acs.biomac.9b01263 31668061

[B7] Ding J. Li C. Zhang Y. Xu W. Wang J. Chen X. (2015). Chirality-mediated polypeptide micelles for regulated drug delivery. Acta Biomater. 11, 346–355. 10.1016/j.actbio.2014.09.043 25278445

[B8] Feng X. Xu W. Xu X. Li G. Ding J. Chen X. (2020). Cystine proportion regulates fate of polypeptide nanogel as nanocarrier for chemotherapeutics. Sci. China Chem. 64 (2), 293–301. 10.1007/s11426-020-9884-6

[B9] Guo H. Li F. Qiu H. Xu W. Li P. Hou Y. (2020). Synergistically enhanced mucoadhesive and penetrable polypeptide nanogel for efficient drug delivery to orthotopic bladder cancer. Research 2020, 1. 10.34133/2020/8970135 PMC7420878 32832909

[B10] Halmos B. Burke T. Kalyvas C. Insinga R. Vandormael K. Frederickson A. (2020). A matching-adjusted indirect comparison of Pembrolizumab plus chemotherapy vs. Nivolumab plus ipilimumab as first-line therapies in patients with PD-L1 TPS >= 1% metastatic NSCLC. Cancers 12 (12), 3648. 10.3390/cancers12123648 PMC7762014 33291810

[B11] Hurwitz H. Fehrenbacher L. Novotny W. Cartwright T. Hainsworth J. Heim W. (2004). Bevacizumab plus irinotecan, fluorouracil, and leucovorin for metastatic colorectal cancer. N. Engl. J. Med. 350 (23), 2335–2342. 10.1056/NEJMoa032691 15175435

[B12] Jang J. Y. Kim H.-J. Han B. W. (2019). Structural basis for the regulation of PPARγ activity by imatinib. Molecules 24 (19), 3562. 10.3390/molecules24193562 PMC6803859 31581474

[B13] Jiang Z.-y. Feng X.-r. Xu W.-g. Zhuang X.-l. Ding J.-x. Chen X.-s. (2020). Calcium phosphate-cured nanocluster of poly(L-glutamic acid)-cisplatin and arsenic trioxide for synergistic chemotherapy of peritoneal metastasis of ovarian cancer. Acta Polym. Sin. 51 (8), 901–910. 10.11777/j.issn1000-3304.2020.20053

[B14] Jung G. Benítez-Ribas D. Sánchez A. Balaguer F. (2020). Current treatments of metastatic colorectal cancer with immune checkpoint inhibitors-2020 update. JCM 9 (11), 3520. 10.3390/jcm9113520 PMC7694085 33142689

[B15] Liu X.-D. Hoang A. Zhou L. Kalra S. Yetil A. Sun M. (2015). Resistance to antiangiogenic therapy is associated with an immunosuppressive tumor microenvironment in metastatic renal cell carcinoma. Cancer Immunol. Res. 3 (9), 1017–1029. 10.1158/2326-6066.Cir-14-0244 26014097 PMC4561186

[B16] Liu Y. Li D. Ding J. Chen X. (2020). Controlled synthesis of polypeptides. Chin. Chem. Lett. 31 (12), 3001–3014. 10.1016/j.cclet.2020.04.029

[B17] Ma W. Chen Q. Xu W. Yu M. Yang Y. Zou B. (2021). Self-targeting visualizable hyaluronate nanogel for synchronized intracellular release of doxorubicin and cisplatin in combating multidrug-resistant breast cancer. Nano Res. 14 (3), 846–857. 10.1007/s12274-020-3124-y

[B18] Patel S. P. Kurzrock R. (2015). PD-L1 expression as a predictive biomarker in cancer immunotherapy. Mol. Cancer Ther. 14 (4), 847–856. 10.1158/1535-7163.Mct-14-0983 25695955

[B19] Ribas A. Tumeh P. C. (2014). The future of cancer therapy: selecting patients likely to respond to PD1/L1 blockade. Clin. Cancer Res. 20 (19), 4982–4984. 10.1158/1078-0432.Ccr-14-0933 24970841 PMC4184978

[B20] Sauer R. Liersch T. Merkel S. Fietkau R. Hohenberger W. Hess C. (2012). Preoperative versus postoperative chemoradiotherapy for locally advanced rectal cancer: results of the German CAO/ARO/AIO-94 randomized phase III trial after a median follow-up of 11 years. JCO 30 (16), 1926–1933. 10.1200/jco.2011.40.1836 22529255

[B21] Skarderud M. R. Polk A. Vistisen K. K. Larsen F. O. Nielsen D. L. (2018). Efficacy and safety of regorafenib in the treatment of metastatic colorectal cancer: a systematic review. Cancer Treat. Rev. 62, 61–73. 10.1016/j.ctrv.2017.10.011 29175677

[B22] Song W. Shen L. Wang Y. Liu Q. Goodwin T. J. Li J. (2018). Synergistic and low adverse effect cancer immunotherapy by immunogenic chemotherapy and locally expressed PD-L1 trap. Nat. Commun. 9, 2237. 10.1038/s41467-018-04605-x 29884866 PMC5993831

[B23] Sun Y. Shan H. Wang J. Wang X. Yang X. Ding J. (2019). Laden nanofiber capsules for local malignancy chemotherapy. J Biomed. Nanotechnol. 15 (5), 939–950. 10.1166/jbn.2019.2745 30890226

[B24] Tokumaru Y. Matsuhashi N. Takahashi T. Tanahashi T. Matsui S. Imai H. (2019). Efficacy of combination therapy with zoledronic acid and cetuximab for unresectable rectal cancer with bone metastases: a case report. Mol. Clin. Onc 10 (6), 571–574. 10.3892/mco.2019.1836 PMC6482389 31031973

[B25] Verschraegen C. F. Jerusalem G. McClay E. F. Iannotti N. Redfern C. H. Bennouna J. (2020). Efficacy and safety of first-line avelumab in patients with advanced non-small cell lung cancer: results from a phase Ib cohort of the JAVELIN Solid Tumor study. J. Immunother. Cancer 8 (2), e001064. 10.1136/jitc-2020-001064 32907924 PMC7481079

[B26] Wang J. Xu W. Li S. Qiu H. Li Z. Wang C. (2018). Polylactide-cholesterol stereocomplex micelle encapsulating chemotherapeutic agent for improved antitumor efficacy and safety. J Biomed. Nanotechnol 14 (12), 2102–2113. 10.1166/jbn.2018.2624 30305217

[B27] Weiser M. R. Quah H.-M. Shia J. Guillem J. G. Paty P. B. Temple L. K. (2009). Sphincter preservation in low rectal cancer is facilitated by preoperative chemoradiation and intersphincteric dissection. Ann. Surg. 249 (2), 236–242. 10.1097/SLA.0b013e318195e17c 19212176

[B28] Wilhelm S. M. Dumas J. Adnane L. Lynch M. Carter C. A. Schütz G. (2011). Regorafenib (BAY 73-4506): a new oral multikinase inhibitor of angiogenic, stromal and oncogenic receptor tyrosine kinases with potent preclinical antitumor activity. Int. J. Cancer 129 (1), 245–255. 10.1002/ijc.25864 21170960

[B29] Zak K. M. Grudnik P. Magiera K. Dömling A. Dubin G. Holak T. A. (2017). Structural biology of the immune checkpoint receptor PD-1 and its ligands PD-L1/PD-L2. Structure 25 (8), 1163–1174. 10.1016/j.str.2017.06.011 28768162

[B30] Zhang H. Dong S. Li Z. Feng X. Xu W. Tulinao C. M. S. (2020). Biointerface engineering nanoplatforms for cancer-targeted drug delivery. Asian J. Pharm. Sci. 15 (4), 397–415. 10.1016/j.ajps.2019.11.004 32952666 PMC7486517

[B31] Zhang W. Ning C. Xu W. Hu H. Li M. Zhao G. (2018). Precision-guided long-acting analgesia by hydrogel-immobilized bupivacaine-loaded microsphere. Theranostics 8 (12), 3331–3347. 10.7150/thno.25276 29930733 PMC6010997

[B32] Zheng P. Liu Y. Chen J. Xu W. Li G. Ding J. (2020). Targeted pH-responsive polyion complex micelle for controlled intracellular drug delivery. Chin. Chem. Lett. 31 (5), 1178–1182. 10.1016/j.cclet.2019.12.001

