# Supplementary material for: Molecular Targeted Agent and Immune Checkpoint Inhibitor Co-Loaded Thermosensitive Hydrogel for Synergistic Therapy of Rectal Cancer
Source: Front Pharmacol. 2021 Apr 16;12:671611. doi: 10.3389/fphar.2021.671611 (PMC8085774; doi:10.3389/fphar.2021.671611)
Supplement: Supplementary file 1 [file datasheet1.docx]

***Supplementary Material***

**Molecular Targeted Agent and Immune Checkpoint Inhibitor Co-loaded Thermosensitive Hydrogel for** **Synergistic Therapy of Rectal Cancer**

Huaiyu Zhang^1^, Jiayu Zhang^1^, Yilun Liu^1^, Yang Jiang^1*^, Zhongmin Li^1*^

^1^ Department of Gastrointestinal Colorectal and Anal Surgery, China-Japan Union Hospital of Jilin University, 126 Xiantai Street, Changchun 130033, P. R. China

*Correspondance:

Yang Jiang

jiangyang@jlu.edu.cn

Zhongmin Li

lizhongmin1211@jlu.edu.com

**Supplementary Figures**


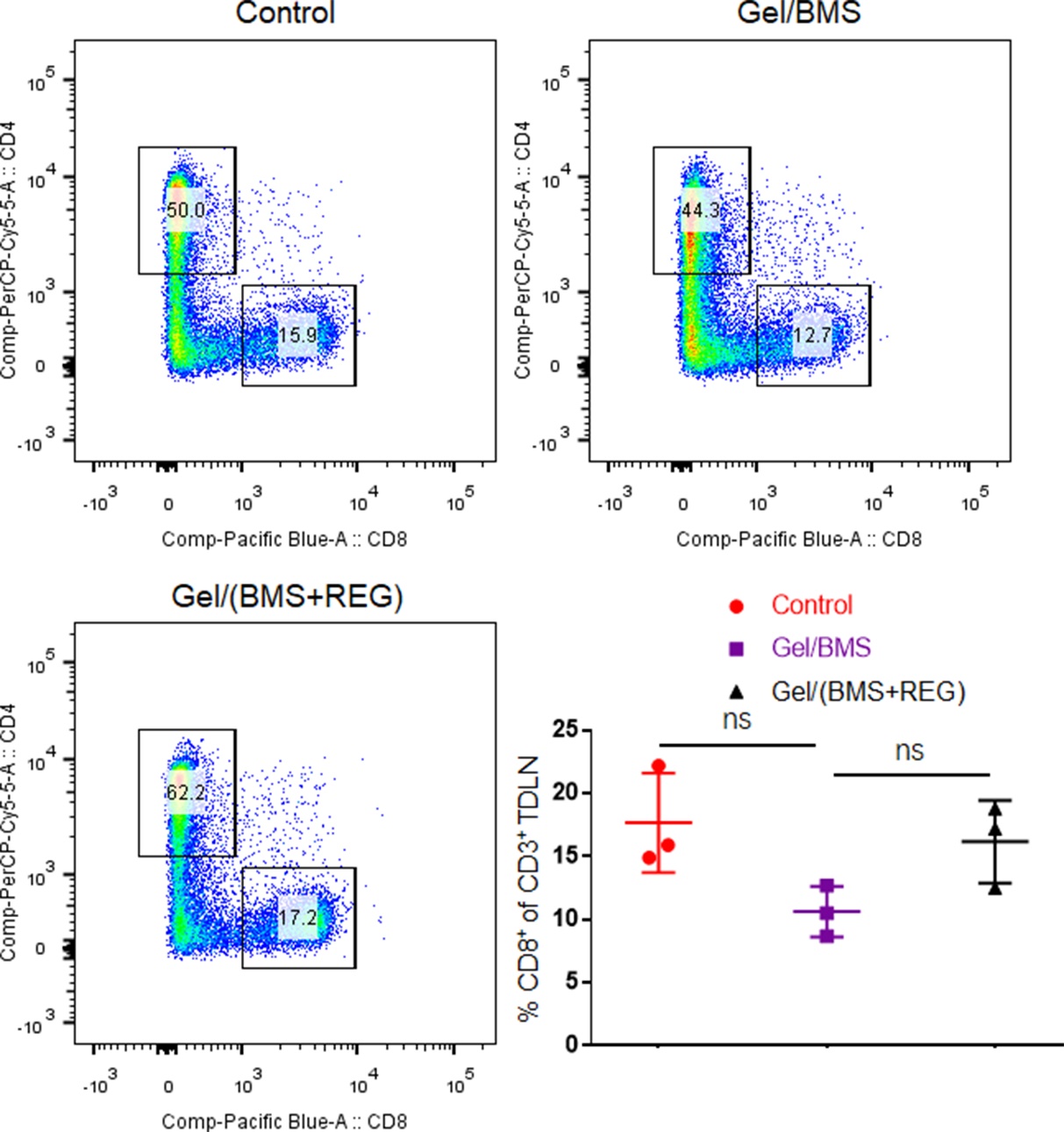


**Supplemental Figure 1.** Flow cytometry analysis of CD8^+^ T cell ratio in TDLN of PBS, Gel/BMS, and Gel/(BMS+REG) groups (*n* = 3).


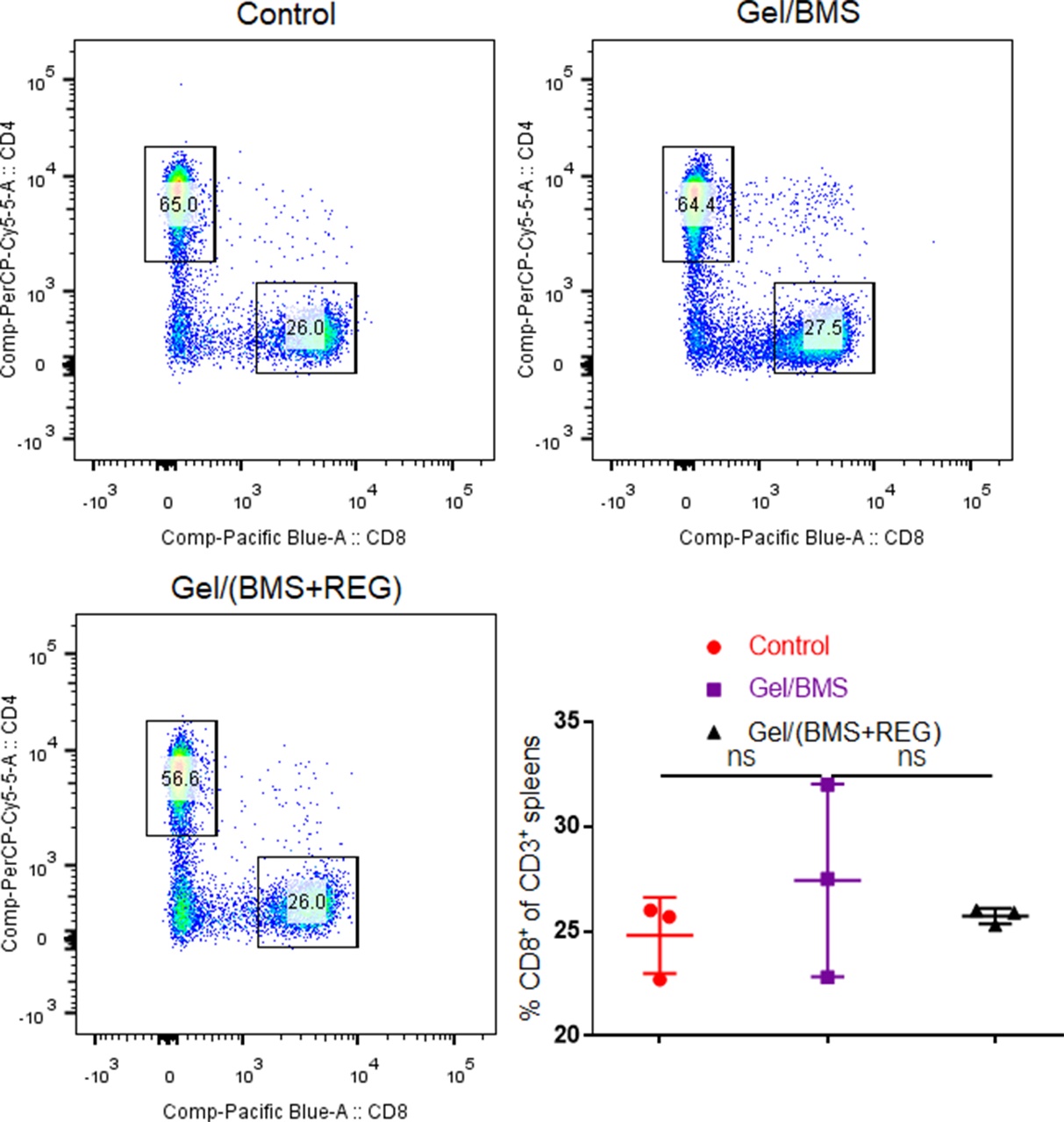


**Supplemental Figure 2.** Flow cytometry analysis of CD8^+^ T cell ratio in spleens of PBS, Gel/BMS, and Gel/(BMS+REG) groups (*n* = 3).


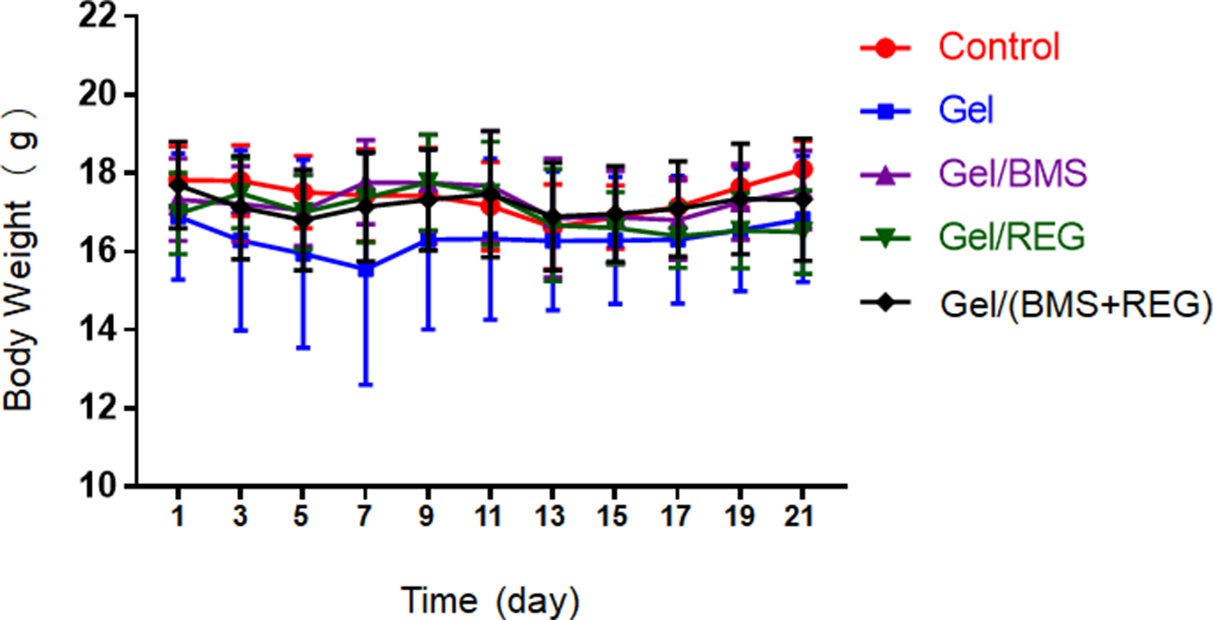


**Supplemental Figure 3.** Measurement of average body mass for each group.
